# Chemical Composition and Antioxidant Activity of Propolis Prepared in Different Forms and in Different Solvents Useful for Finished Products

**DOI:** 10.3390/foods7030041

**Published:** 2018-03-19

**Authors:** Fabio Galeotti, Francesca Maccari, Alfredo Fachini, Nicola Volpi

**Affiliations:** 1 Department of Life Sciences, University of Modena & Reggio Emilia, Via Campi 213/D, 41121 Modena, Italy; fabio.galeotti@unimore.it (F.G.); francesca.maccari@unimore.it (F.M.); 2 B Natural, Corbetta, 20121 Milano, Italy; alfredo.fachini@bnatural.it

**Keywords:** propolis, polyphenols, flavonoids, phenolic acids, European brown poplar propolis

## Abstract

Different products from a unique propolis extract obtained by using various solvents such as hydroalcoholic, glycolic (98% propylene glycol), and glyceric solutions, and oil, as well as in powder form, named ESIT12, were prepared. The molecular composition of the different preparations was evaluated and their antioxidant activity determined. All the preparations showed a quite similar polyphenol composition and comparable percentage even if ESIT12 was found to be richer in phenolic acids (caffeic, coumaric, ferulic, and isoferulic). Overall, flavones and flavonols ranged from ~20% up to ~36% in the glyceric extract, while flavanones and diidroflavonols were between ~28% and ~41%. Besides their quite similar composition, glycolic and hydroalcoholic extracts were found to be richer in the total polyphenols content. When the antioxidant properties were determined for the four preparations, the activity was similar among them, thus revealing that it is strictly related to the polyphenols content for propolis products whose composition is quite comparable. To date, very few data are available on propolis composition in glyceric and glycolic extracts and information has never been published on propolis in oil. This study could be of interest to the food and nutraceutical industries to choose suitable solvents and conditions to produce propolis preparations useful for active finished products.

## 1. Introduction

Propolis is a complex material of resinous consistency produced by bees which has a highly variable physical appearance, color, and consistency, depending on many factors such as geographic origin, types of vegetable sources, time of collection, and season of the year [1]. Bees use propolis to seal openings in the hive to avoid the entrance of intruders, to maintain a constant inner temperature, to contribute to the attainment of an internal aseptic environment, and overall to protect the hive from widespread bacterial infection [1,2,3,4]. Propolis composition is extremely complex and variable, showing the presence of beeswax, resin, essential oils, and pollen. Bees secrete the wax, while resin and oils are obtained from plants, usually taken from secretions or by cutting fragments of vegetative tissues.

The variations in the chemical composition and consequently in the biological activity of propolis, are associated with its type and geographic origin. However, although propolis is a complex mixture, its biological activities are reported due to the presence of the flavonoids, phenolic acids, and ethers mainly obtained from plant-derived substances [5]. Hence, although propolis is obviously an animal product, a considerable proportion of its components responsible for biological activities are plant derived. In fact, the resin contains most of the compounds found in extracts consumed by people from many countries as food complements, nutraceuticals, or alternative medicine. In fact, propolis has been extensively used in folk medicine for many years, and there is substantial evidence to indicate that it has antiseptic, antifungal, antibacterical, antiviral, anti-inflammatory, and antioxidant properties [4,5,6,7]. Current applications of propolis include over-the-counter preparations for cold syndrome (upper respiratory tract infections, common cold, flu-like infection), as well as dermatological preparations useful in wound healing, treatment of boils, acne, herpes simplex and genitalis, and neurodermatitis, among other ailments [4,8]. These effects are exerted by the numerous, more than 300, known substances composing propolis [9].

Propolis, which is barely soluble in water, cannot be used as a raw material and it must be purified by extraction with solvents to remove the inert material and preserve the polyphenolic fraction. These last compounds, flavonoids and phenolic acids, are considered to contribute more to the healing effects than the other propolis constituents [9]. Propolis extracts are more commonly obtained through conventional techniques, such as ethanolic or aqueous extraction or by Soxhlet [10,11]. In fact, in commercial and supplemental health care products, propolis is added in the form of extracts which are obtained by soaking crushed propolis in organic solvent or water. Moreover, in the last few years, different studies have shown the extraction with supercritical fluid as a possible alternative method to obtain compounds derived from natural matrices, including propolis [10].

Due to the very high molecular complex composition of propolis, the different extractive processes utilized and the various solvents available to produce the finished products, the evaluation of the total polyphenols extracted, as well as an accurate determination of the single species and of the derived families of compounds with similar chemical properties, are necessary to assure products rich in molecular species and still retaining biological properties. In fact, despite the high number of compounds extracted, the yield of the extraction process is often lower, which could indicate a selectivity depending on the extractive process. Moreover, the different solvents used to solubilize the extracted polyphenols from propolis may have a selective capacity to produce finished products which possess peculiar compositions and biological properties.

In this paper, we evaluated this aspect by dissolving a unique propolis extract in various solvents obtaining different finished products such as hydroalcoholic, glycolic, glyceric solutions, and oil, as well as a product in the form of powder. The molecular composition of the different preparations was evaluated by high-performance liquid chromatography-UV-electrospray ionization mass (HPLC-UV-ESI-MS) and the antioxidant activity determined by a 2,2-diphenyl-1-picrylhydrazyl (DPPH) anti-oxidant assay [12]. This is more important by considering that the finished products in various forms often require propolis dissolved in specific solvents and supporting matrixes. Finally, up to now, very few data are available on propolis composition in glyceric and glycolic extracts and no information has ever been published on propolis in oil.

## 2. Materials and Methods

### 2.1. Materials

Finished samples from the same poplar propolis extract were prepared by B Natural (Corbetta, Milano, Italy, http://www.bnatural.it/) consisting of one powder product named ESIT12 and four liquid extracts. ESIT12 is a micronized sample composed of propolis with a minimum of 12% total polyphenols supported by sucrose and silicon dioxide. The four liquid samples were prepared by B Natural in hydroalcoholic (80% ethanol/20% water), glycolic (98% propylene glycol with the remaining 2% of ethanol), and glyceric (95% and 5% water) solutions, and oil (100% seed oil).

Various phenolic acids and flavonoids used as standards were purchased from Sigma-Aldrich (http://www.sigmaaldrich.com/).

### 2.2. Extraction of Polyphenols for HPLC-UV-ESI-MS Analysis and Assays

A total of 500 mg of ESIT12 was dissolved in 50 mL (10 mg/mL) ethanol/water 80%/20%. After vigorous mixing and sonication for 10 min, polyphenols were extracted at 70 °C in a water bath for a minimum of 2 h under continuous mixing. After centrifugation at 10,000 RPM for 10 min, the sample was further analyzed according to the methodologies illustrated below. Liquid samples were analyzed with no further modification.

### 2.3. HPLC-UV-ESI-MS

The high-performance liquid chromatography equipment was from Jasco (Jasco, Tokyo, Japan) (pump mod. PU-1580, UV detector UV-1570, Rheodyne injector equipped with a 20 µL loop, software Jasco-Borwin rel. 1.5). The phenolic acids and flavonoids from propolis were separated using a 250 × 4.6-mm stainless-steel column Discovery-C18 4 µm 80 Ä (from Sigma-Aldrich). The eluents were (A) 0.5% acetic acid and (B) acetonitrile. Separations were performed at room temperature by solvent gradient elution from 0 min at 50% A/50% B to 60 min at 100% B at a flow rate of 0.8 mL/min. A UV detector set at 260 nm was also used on-line with HPLC equipment.

An Agilent 1100 VL series mass spectrometer (Agilent Technologies, Inc., Santa Clara, CA, USA) was further used on-line with HPLC equipment. The electrospray interface was set in negative ionization mode with the capillary voltage at 3500 V and a temperature source of 350 °C in full scan spectra (200–2200 Da, 10 full scans/s). Nitrogen was used as a drying (9 L/min) and nebulizing gas (11 p.s.i.). Software versions were 4.0 LC/MSD trap control 4.2 and Data Analysis 2.2 (Agilent Technologies, Inc.).

Additionally, 2–20 µL of samples were injected at a standardized concentration expressed as the total polyphenol content evaluated by a spectrophotometric assay according to Folin-Ciocalteau (see below) against a calibration curve performed by pure galangin.

### 2.4. Total Polyphenol Content

Phenolic compounds are oxidized by Folin-Ciocalteau reactive (Sigma-Aldrich) and the blue colour produced presents a maximum absorption at 750 nm evaluated by a spectrophotometer against a specific blank. Briefly, 20 µL of the propolis extracts was added to 380 µL of ethanol and 100 µL of the Folin-Ciocalteau reagent. Then, 200 µL of a saturated sodium carbonate solution and 4.3 mL water were added. The tubes were then allowed to stand at room temperature for 60 min before measuring the absorbance at 750 nm against the blank. The total polyphenolic content was expressed as galangin equivalents in % *w*/*w* or mg/mL. The concentration of polyphenols in the samples was derived from a standard curve of galangin ranging from 5 to 60 µg (correlation coefficient > of 0.9890).

### 2.5. Antioxidant Activity

The radical scavenging effect on 2,2-diphenyl-1-picrylhydrazyl (DPPH) molecules was evaluated according to the method of Banskota and others [12] with slight modifications. Samples were dissolved in a buffer solution at a final concentration of 1 μg/mL or final dilution of 1 μL/mL. For each extract, a series of test tubes were prepared containing between 1 and 1000 μL of these solutions, and the volume completed to 1000 μL with buffer. Finally, 1 mL of a 500 μM DPPH solution was added to each tube. After 30 min of incubation at room temperature in the dark, the absorbance was recorded at 517 nm by a spectrophotometer. Results are expressed in terms of the percentage of decrease with respect to control values (absorbance of 1 mL DPPH solution + 1 mL of buffer) incubated under the same conditions. Readings were made in triplicate. The mean of each result is plotted on a graph constructed by plotting increasing concentrations of the antioxidant trolox (6-hydroxy-2,5,7,8-tetramethylchroman-2-carboxylic acid) vs. Optical Density (OD) values. Propolis samples’ antioxidant activity is expressed as µg trolox equivalent.

## 3. Results

**Figure 1:**
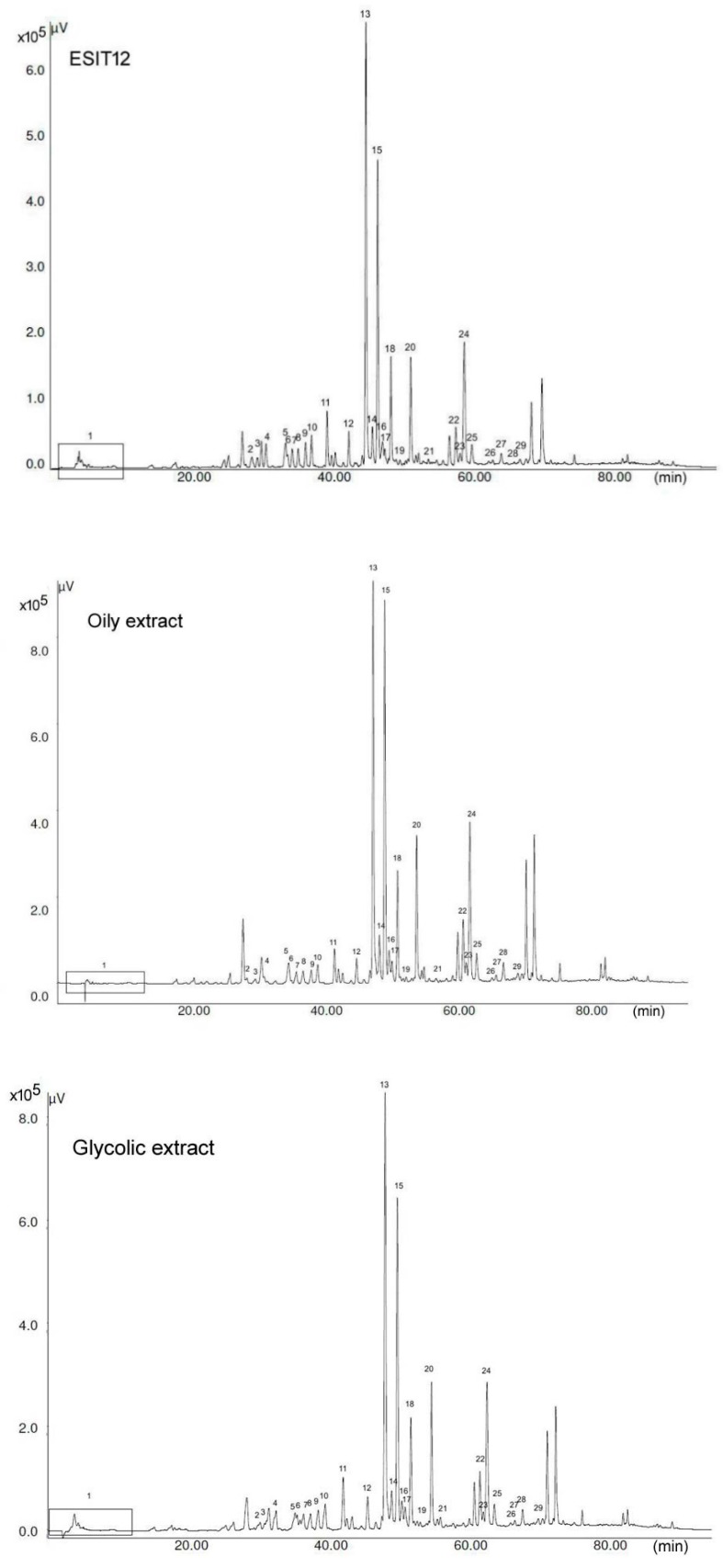

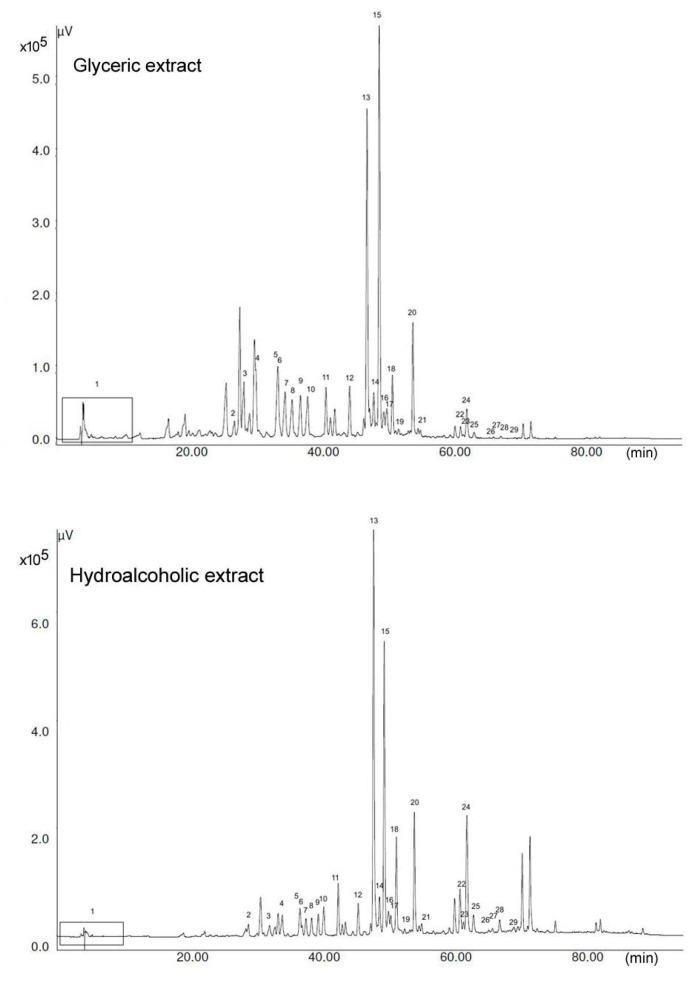
High-performance liquid chromatography-UV (HPLC-UV) profile of the various propolis preparations. Polyphenols species are numbered accordingly and listed in Table 1.

**Figure 2:**
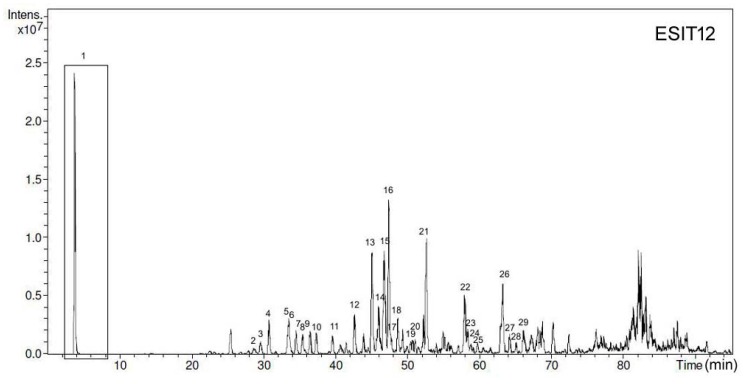

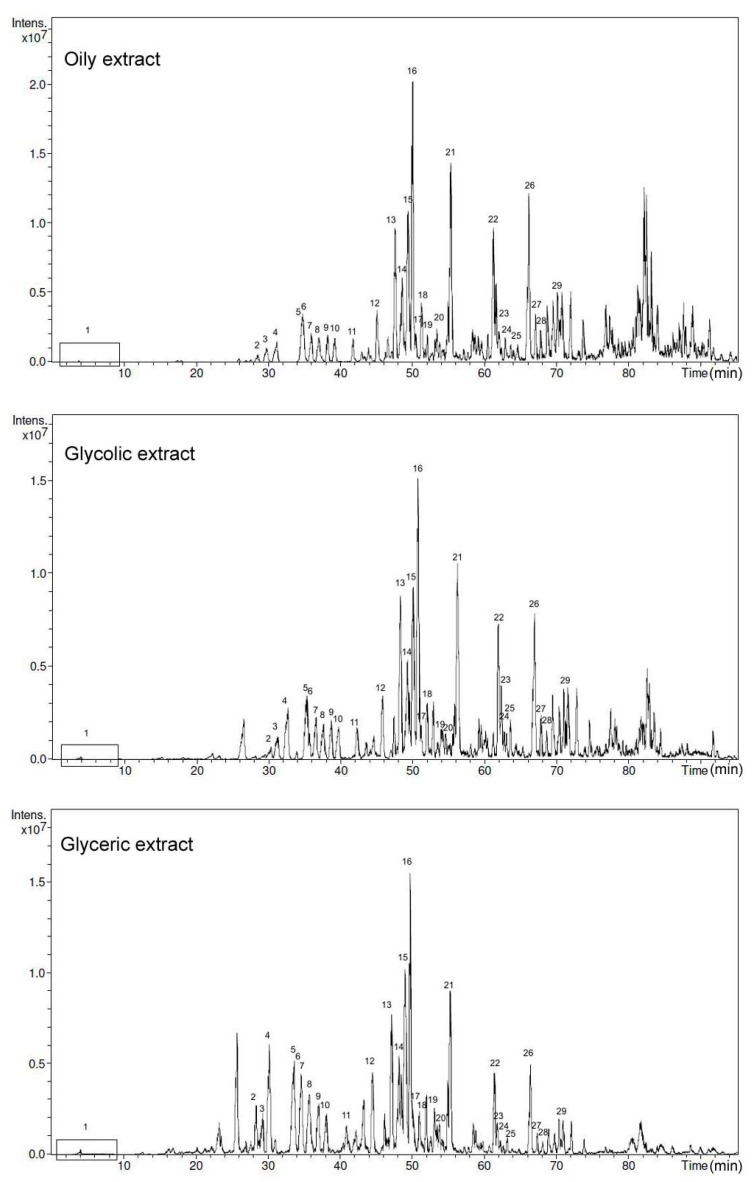

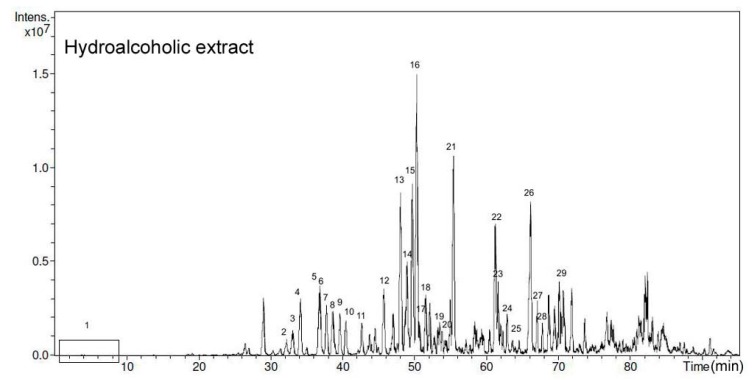
HPLC-MS chromatogram of the various propolis preparations. Polyphenols species are numbered accordingly and listed in Table 1.

HPLC-UV-ESI-MS and tandem MS analyses [13] of the various propolis preparations in various solvents led to the identification of ~30 compounds in each sample (Table 1), accounting for ~60–70% of the total molecular species. Identification was performed through the comparison of chromatographic retention times, and MS and tandem MS spectra, with the literature and with commercially available standard compounds, where possible. UV acquired at 260 nm and MS profiles are illustrated in Figure 1 and Figure 2, respectively, for the different propolis extracts. Table 1 also reports the quantitative data obtained with a UV detector by using a real commercially available standard or standard compounds possessing quite similar spectroscopic properties (for example, for quantitation of the various pinobanksin derivatives). The remaining unidentified part could be mainly represented by the family of triterpenoids and a small fraction of glycosylated derivatives [11]. In fact, triterpenoids are abundant and very common in all vegetable forms and they are generally present in any kind of extract [14].

ESIT12 is a micronized product composed of propolis extract from a raw sample to produce a highly pure propolis free of wax and other possible contaminants supported by sucrose and silicon dioxide. The other four extracts were produced from the same propolis sample of ESIT12 but in different solvents, hydroalcoholic (80% ethanol/20% water), glycolic (98% propylene glycol), and glyceric solutions, and seed oil. As evident from Table 1, besides their different preparation and solvents, all these extracts show a quite similar polyphenol composition, as well as a comparable percentage. ESIT12 is indeed richer in phenolic acids (caffeic, coumaric, ferulic, and isoferulic) than liquid preparations, ~10% vs. 0.5% (Table 1), mainly due to the specific and owner production procedure. Apart from this difference, all five extracts show the presence of pinobanksin, chrysin, pinocembrin, galangin, pinobanksin-3-*O*-acetate, pinobanksin-3-*O*-butyrate, and various other pinobanksin derivatives such as the main bioflavonoid species. Overall, flavones and flavonols range from ~20% up to ~36% in the glyceric extract, while flavanones and diidroflavonols are between ~28% and ~41% (Table 1).

Besides their quite similar composition, the total polyphenols content, phenolic acids and bioflavonoids, was different for the various preparations (Table 2). In fact, glycolic and hydroalcoholic extracts were found to contain a greater polyphenol percentage (Table 2). This is probably due to the capacity of different solvents to solubilize more species than others when using the same starting raw propolis for all preparations and a quite similar extractive process (not shown, an owner process of B Natural). In fact, the well-known high capacity of hydroalcoholic solution, generally 80% ethanol and 20% water, to solubilize a great percentage of polyphenols [10,11,13] was also confirmed in this study. Moreover, we also measured a high capacity of the glycolic solvent to solubilize a very high % of polyphenols with a quite similar composition when other solvents are used. This may be of some importance for finished products produced by using glycolic preparations of propolis. However, the antioxidant activity expressed as µg trolox equivalent/mg polyphenol was found to be quite similar for the various preparations, showing that all samples are able to exert antioxidant capacity and that this activity is strictly related to the polyphenols content for propolis products with a quite similar composition (Table 2).

## 4. Discussion

Propolis is largely used in drinks and foods in the form of liquids, effervescient, tablets, pills, and others as an ingredient in many dietary supplements and nutraceuticals for human nutrition to prevent diseases and improve health [4,5,6]. In fact, propolis is used alone or in combination with other natural products, not only as a nutraceutical supplement, but also as a natural antioxidant in food and related products [15]. Nutraceuticals in particular have emerged in these last years as a major consumer-driven trend capable of preventing diseases, delaying aging, and enhancing well-being and performance [16,17]. As it is evident, scientific information on the composition, structure, and biological activity of nutraceuticals is very important for the advancement of their knowledge. As a consequence, considering the well-known propolis properties and its huge fields of application, there is an increased interest in its activities and composition in the food and nutraceutical products.

The nature of the solvent, temperature, and time of treatment, as well as composition and physical characteristics of propolis samples, strongly influence the yield of extraction and quality of final products [10,11]. By maintaining the time and keeping the temperature constant, the polarity of the solvent and propolis composition are the two most important factors able to influence the quality of extracts. By using the same propolis extract, five different finished products were produced; one in powder form and the other four as liquid solutions. ESIT12 is a micronized sample containing a minimum of 12% total propolis polyphenols used in the preparation of tablets and capsules. Glycolic, glyceric, and oily extracts are largely used in various liquid products as nutraceuticals, and hydroalcoholic preparation was prepared due to its large application in the most common commercial products from propolis. Total phenolics and bioflavonoid components (flavones and flavonols, flavanones, and dihydroflavonols) were determined by HPLC-UV-ESI-MS and tandem mass and further characterized for antioxidant activity. In fact, according to its European brown poplar propolis origin, the raw propolis used to obtain the various preparations was found to possess flavonoids as the major species, with flavones (i.e., chrysin and apigenin), flavanones (pinocembrin), and flavonols (quercetin, pinobanksin, galangin, and their derivatives) being the most common components [6]. Up to now, very few data are available on glyceric and glycolic propolis preparations and no information has ever been published on propolis in oil, as well as propolis in powder form like ESIT12. As a consequence, it is not possible to make a comparison of the composition and activity with other information available in scientific literature. As reported in our study, we demonstrate that propolis solubilized in different solvents has a similar chemical composition when produced from the same raw material, even if a different total polyphenol percentage is obtained, i.e., more or less rich in polyphenol content. A direct consequence of the same composition is a quite similar antioxidant activity when calculated for mg of polyphenols. On the other hand, finished products may have different biological activity if extracted from propolis which has a different polyphenolic composition and quality [18]. In fact, the health-promoting capacities of propolis mainly depend on the phenolic compounds and content which represent its biologically active component, as also reported for other kind of extracts [19]. Finally, this study could be of interest for the food and nutraceutical industries choosing suitable solvents and conditions to solubilize propolis extracts useful to prepare active finished products.

## 5. Conclusions

In this study, we demonstrated that propolis solubilized in different solvents and in liquid and solid form has a similar chemical composition when produced from the same raw material. To this aim, we prepared hydroalcoholic, glycolic (98% propylene glycol), glyceric and oily solutions as well as in powder form, named ESIT12, from the same propolis extract. A direct consequence of the same composition is a quite similar antioxidant activity when calculated for mg of polyphenols so we can produce finished products having a defined composition-activity relationship.

## Figures and Tables

**Table 1:** Molecular composition of the various propolis preparations determined by high-performance liquid chromatography-UV-electrospray ionization mass (HPLC-UV-ESI-MS).

No.	Polyphenols Species	ESIT12	Oily Extract	Glycolic Extract	Glyceric Extract	Hydroalcoholic Extract
		% *w*/*w*	% *w*/*v*	% *w*/*v*	% *w*/*v*	% *w*/*v*
1	Phenolic acids (caffeic, coumaric, ferulic, isoferulic)	9.7	0.5	0.4	0.5	0.5
2	Quercetin	0.4	0.3	0.5	1.5	0.5
3	Pinobanksin 5-methyl ester	0.6	0.7	1.0	1.4	0.9
4	Quercetin 3-methyl ester	1.8	1.0	2.4	5.0	2.3
5	Pinobanksin	2.0	2.2	3.1	4.0	2.2
6	Apigenin	0.4	0.5	0.5	1.1	0.5
7	Kaempferol	1.1	1.1	1.6	3.8	1.7
8	Isorhamnetin	1.1	1.2	1.7	3.3	1.7
9	Luteolin 5-methyl ester	1.1	1.0	1.4	2.1	1.2
10	Quercetin 5,7-dimethyl ester	1.1	0.9	1.3	1.7	1.2
11	Galangin 5-methyl ester	1.0	0.8	1.1	1.1	0.8
12	Quercetin 7-methyl ester	2.0	1.8	2.1	3.2	2.2
13	Chrysin	5.3	4.5	5.4	5.0	5.3
14	Pinocembrin	2.0	3.1	3.1	2.9	3.1
15	Galangin	5.8	5.2	5.9	7.5	6.0
16	Pinobanksin-3-*O*-acetate	6.7	8.4	8.1	9.5	8.0
17	CAPE	0.4	0.4	0.6	0.3	0.4
18	Metoxychrysin	1.6	1.6	1.5	1.1	1.6
19	Pinobanksin-3-*O*-propionate	0.3	1.0	1.1	1.2	0.6
20	Caffeic acid cinnamyl ester	0.4	0.3	0.3	0.2	0.3
21	Pinobanksin-3-*O*-butyrate	5.4	6.5	6.7	5.7	6.7
22	Pinobanksyn-3-*O*-pentenoate	3.0	4.1	3.8	2.6	4.2
23	Other Pinobanksin derivative	1.1	1.7	1.6	0.7	1.7
24	Pinobanksin-3-*O*-hexanoate	0.3	0.7	0.4	0.3	0.5
25	Other Pinobanksin derivative	0.2	0.6	0.8	0.4	0.4
26	Other Pinobanksin derivative	4.5	6.0	5.0	3.3	5.8
27	Other Pinobanksin derivative	0.8	1.2	0.9	0.5	1.0
28	Other Pinobanksin derivative	0.4	0.6	0.5	0.2	0.7
29	Other Pinobanksin derivative	1.2	4.5	1.3	0.7	3.7
	Total identified polyphenols	61.7	62.4	64.1	70.8	65.7
	Phenolic acids and derivatives	10.5	1.2	1.3	1.0	1.2
	Flavones and flavonols	22.7	19.9	25.4	36.4	25.0
	Flavanones and dihydroflavonols	28.5	41.3	37.4	33.4	39.5

**Table 2:** Polyphenols content and antioxidant activity of the various propolis preparations measured by the radical scavenging effect on 2,2-diphenyl-1-picrylhydrazyl (DPPH) and reported as µg trolox (6-hydroxy-2,5,7,8-tetramethylchroman-2-carboxylic acid) equivalent/mg polyphenols. Data are illustrated as mean ± standard errors.

Propolis Finished Products	Polyphenols Content	Microg Trolox/mg Polyphenols
ESIT12 (*w*/*w*)	16.5 ± 0.8%	74.0 ± 4.2%
Oily extract (*w*/*v*)	24.0 ± 1.4%	79.4 ± 4.2%
Glycolic extract (*w*/*v*)	81.2 ± 3.7%	71.7 ± 3.5%
Glyceric extract (*w*/*v*)	26.2 ± 1.6%	74.4 ± 3.8%
Hydroalcoholic extract (*w*/*v*)	69.7 ± 2.0%	76.0 ± 4.1%
